# Association Between First-Trimester Serum PAPP-A and Free β-hCG Levels and Gestational Duration: A Retrospective Cohort Study

**DOI:** 10.1007/s11845-026-04493-x

**Published:** 2026-06-16

**Authors:** Gorkem Arica, Dogu Kucuksuleymanoglu, Ismail Yilmaz, Merve Aytac, Karolin Ohanoglu, Ebru Alici Davutoglu, Dildar Konukoglu, Riza Madazli

**Affiliations:** 1 Cerrahpasa Faculty of Medicine, Department of Obstetrics and Gynecology, Istanbul University-Cerrahpasa, Istanbul, Turkey; 2 Department of Obstetrics and Gynecology, Istanbul Cam and Sakura City Hospital, Istanbul, Turkey; 3 Cerrahpasa Faculty of Medicine, Department of Biochemistry, Istanbul University-Cerrahpasa, Istanbul, Turkey; 4 Department of Obstetrics and Gynecology, Division of Perinatology, Cerrahpasa Medical Faculty, Istanbul University–Cerrahpasa, Kocamustafapasa, St. No. 53, Istanbul, 34098 Turkey

**Keywords:** PAPP-A, Gestational duration, First-trimester screening, Placental function

## Abstract

**Background:**

Gestational duration is influenced by multiple factors, with placental function being one of the key determinants. First-trimester serum biomarkers reflect the quality of placentation and may be associated with the duration of pregnancy.

**Aims:**

To investigate the association between first-trimester serum placental biomarkers and gestational duration, and to evaluate the independent effects of nulliparity and fetal sex on gestational length.

**Methods:**

This retrospective, single-center study included 346 singleton pregnancies delivered between 34 + 0 and 41 + 0 weeks of gestation following spontaneous labor. First-trimester PAPP-A and free β-hCG multiples of the median (MoM) values were measured. Gestational length was calculated as the interval between the estimated last menstrual period and the date of delivery. Spearman correlation analysis and multiple linear regression were used to assess associations with gestational duration.

**Results:**

The mean gestational duration was 271 ± 10.5 days. Pregnancy duration was significantly longer in the PAPP-A MoM ≥ 0.6 group compared to the < 0.6 group (273 vs. 269 days, *p* = 0.001). In multiple linear regression analysis, PAPP-A MoM (B = 2.08, *p* = 0.034), nulliparity (B = 2.96, *p* = 0.013), and fetal sex (B = 2.63, *p* = 0.022) were identified as independent predictors of gestational duration. No significant association was observed between free β-hCG MoM and gestational duration.

**Conclusions:**

Higher first-trimester PAPP-A MoM levels are independently associated with longer gestational duration, even after adjustment for parity and fetal sex, suggesting that early placental function may play a role in regulating the timing of parturition with modest effect.

## Introduction

Despite significant advances in medical science, the precise biological mechanisms that govern the timing of parturition remain poorly understood. While mammals exhibit species-specific pregnancy durations, human gestation is conventionally defined as 280 days, though it is characterized by substantial natural variation ranging from 259 to 294 days. Furthermore, calculating gestational age based on the last menstrual period (LMP) introduces considerable variance due to the inherent uncertainty regarding the exact timing of ovulation. Therefore, first-trimester measurement of crown–rump length (CRL) provides a more accurate assessment of true gestational age [1]. Nevertheless, estimation of the due date still relies on the conventional method of arbitrarily selecting the 280th day of gestation and only 4% of pregnant women deliver on their estimated due date [2]. This practice persists even in pregnancies achieved through assisted reproductive technology (ART), where the timing of conception and embryo transfer is precisely known; yet considerable variation in pregnancy duration still exists, which cannot be explained solely by differences in implantation timing [3].

Progesterone withdrawal is a widely postulated theory for the initiation of parturition; however, this concept is primarily based on animal models. Human studies have consistently failed to demonstrate a significant decrease in, or withdrawal of, maternal serum progesterone levels preceding the onset of labor [4]. An alternative hypothesis, known as the ‘energetics of gestation and growth’ (EGG), proposes that the balance between maternal energy supply and fetal metabolic demand determines the timing of birth [5]. This hypothesis underscores the pivotal role of the placenta; supporting this notion, research has shown that a higher placental-to-fetal volume ratio at 27 weeks of gestation is significantly associated with a longer duration of pregnancy [6].

Pregnancy-associated plasma protein-A (PAPP-A) is a metalloproteinase that targets and cleaves insulin-like growth factor binding proteins (IGFBP)−4 and − 5 [7]. Through this proteolytic activity, it regulates the bioavailability of insulin-like growth factors (IGF)−1 and − 2, which are critical for healthy trophoblastic invasion and adequate placental development [7]. These growth factors contribute to cellular proliferation and differentiation, ensuring optimal fetal and placental growth [8]. Consequently, low maternal serum PAPP-A levels serve as clinical indicators of impaired placentation and are significantly associated with an increased risk of adverse perinatal outcomes, including preeclampsia, fetal growth restriction (FGR), and low birth weight [9, 10].

In this study, we hypothesized that lower PAPP-A MoM values may be associated with shorter gestational length and aimed to demonstrate the relationship between the first-trimester placental biomarkers and duration of gestation. We also evaluated the effects of other contributing factors, such as nulliparity and fetal sex.

## Methods

This retrospective, single-center study was conducted in the Department of Maternal-Fetal Medicine at a tertiary university hospital. Ethical approval for the study was obtained from the hospital’s institutional review board (Approval Date: 11/07/2025; Decision no: 175). Due to the retrospective design, the requirement for written informed consent was waived. The study was conducted in accordance with the ethical principles of the Declaration of Helsinki. Data were extracted from medical records and the hospital’s electronic database for pregnant women who underwent first-trimester anatomy screening and combined screening test between January 2022 and January 2025.

Women who were admitted to the labor ward and delivered between 34 + 0 and 41 + 0 weeks of gestation were included. Labor was defined as the presence of effective, spontaneous, regular uterine contractions accompanied by cervical dilation and effacement. Uterine contraction intensity was quantified using Montevideo units, calculated by subtracting the baseline intrauterine pressure from the peak value (in kilopascals) for each contraction and summing all such values recorded within a 10-minute period; a minimum of three to five contractions in 10 min with a total intensity of ≥ 200 Montevideo units was required. Following admission, women with a history of cesarean section underwent repeat cesarean delivery, while the remaining patients were managed for vaginal delivery unless an intrapartum indication for cesarean section arose. Oxytocin was administered when indicated for augmentation in cases of prolonged labor.

Exclusion criteria included multiple pregnancies; pre-existing maternal conditions such as chronic hypertension, diabetes mellitus, and rheumatologic disorders; and pregnancies conceived via in vitro fertilization. Patients with uterine anomalies (e.g., unicornuate, septate, or bicornuate uterus), uterine fibroids, or a history of uterine surgery other than cesarean section were also excluded. Additionally, women with a history of preterm delivery, those who developed preeclampsia, gestational diabetes mellitus, or early-onset fetal growth restriction during the current pregnancy, and fetuses with chromosomal or genetic abnormalities detected prenatally or postnatally were excluded. Pregnancies requiring medically indicated induction of labor were also excluded.

**Fig. 1:**
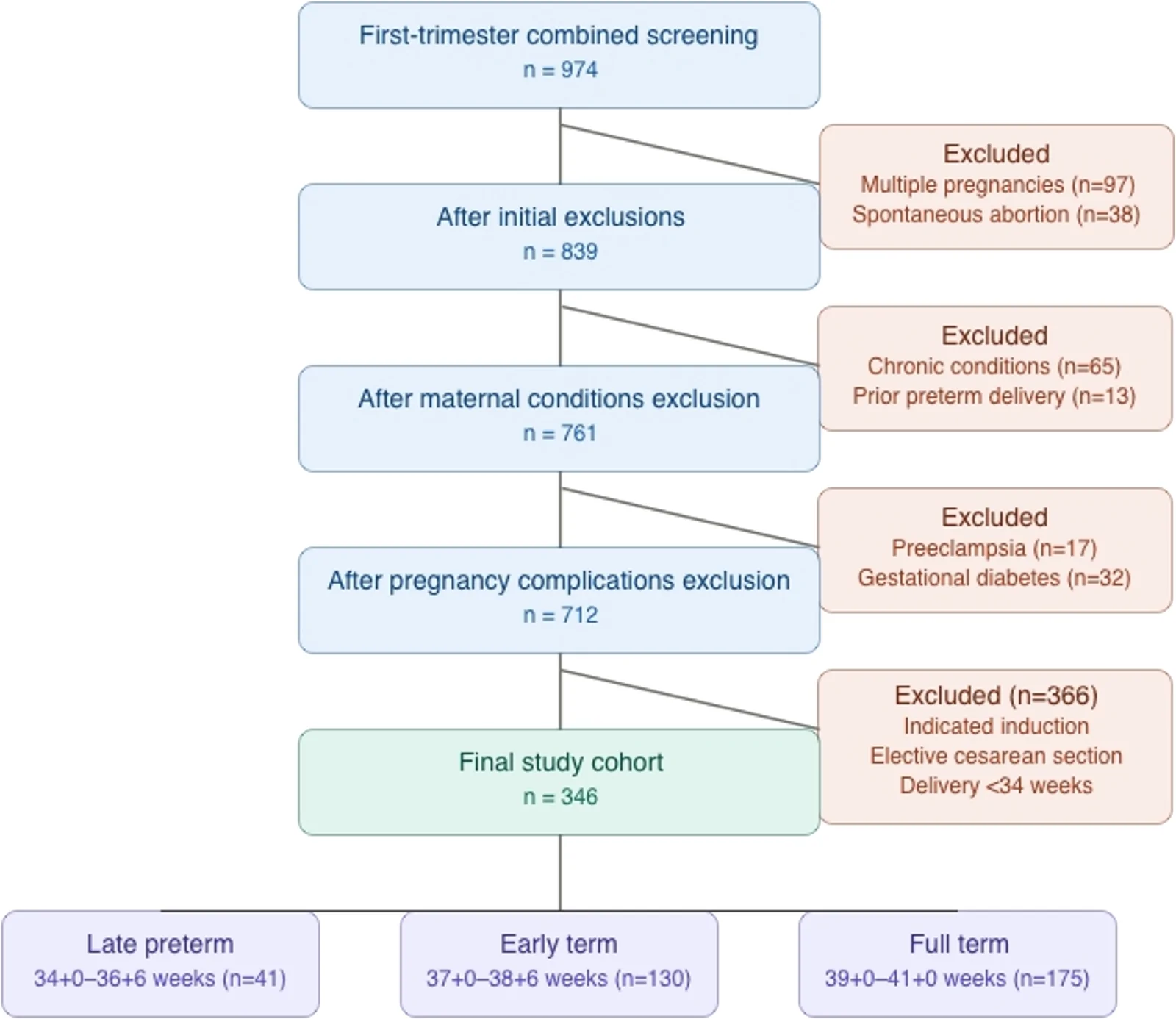
Flowchart of the patient selection for the study

A total of 974 first-trimester combined screening tests were performed during the study period. Of these, 97 involved multiple pregnancies and 38 resulted in spontaneous abortion. Sixty-five women had pre-existing chronic conditions, and 13 had a history of preterm delivery. Seventeen patients developed preeclampsia, and 32 developed gestational diabetes mellitus. An additional 366 patients were excluded due to other criteria, including medically indicated induction of labor, elective cesarean section, and delivery before 34 weeks of gestation. Ultimately, 346 patients were included in the final analysis (Fig. 1).

Gestational age was determined based on first-trimester crown–rump length (CRL) measurements. CRL measurements below 60 mm were used for dating and to estimate the last menstrual period (LMP). All ultrasound examinations were performed by maternal–fetal medicine specialists using Voluson E10 and Voluson S8 ultrasound systems (GE Healthcare, Zipf, Austria), equipped with both curvilinear and transvaginal transducers. Gestational length was calculated as the interval between the estimated LMP and the date of delivery and was expressed in days. Late preterm, early term, and full-term deliveries were defined as births occurring between 34 + 0 and 36 + 6, 37 + 0 and 38 + 6, and 39 + 0 and 41 + 0 weeks of gestation, respectively.

Peripheral venous blood samples were collected and centrifuged for serum separation, and all biochemical analyses were performed on the day of sample collection. Maternal serum PAPP-A and free β-hCG concentrations were measured by electrochemiluminescence immunoassay using the Elecsys PAPP-A and Elecsys free β-hCG assays, respectively (Roche Diagnostics GmbH, Mannheim, Germany). According to the manufacturer’s performance data, the PAPP-A assay showed repeatability (intra-assay) CVs of 1.2%–5.0% and intermediate precision (inter-assay) CVs of 2.0%–6.9%, while the free β-hCG assay showed repeatability CVs of 1.0%–1.6% and intermediate precision CVs of 1.5%–3.8%. Multiples of the median (MoM) values for PAPP-A and free β-hCG were calculated using prenatal screening software (SSDWLAB6; SBP Soft 2007 S.L., Spain), with adjustment for maternal body mass index (BMI), maternal age, sonographic gestational age, smoking status, and fetal nasal bone status.

## Statistical Analysis

Statistical analyses were performed using IBM SPSS Statistics software, version 31 (IBM Corp., Armonk, NY, USA). The distribution of variables was evaluated using the Shapiro-Wilk test. Continuous data are presented as mean ± standard deviation (SD) for normally distributed variables or as median (25th–75th percentile) for non-normally distributed variables.

For comparisons between two groups, the independent samples t-test or Mann-Whitney U test was employed, as appropriate. Comparisons across three groups were conducted using one-way ANOVA or the Kruskal-Wallis test. Following a significant result in the three-group analysis, post-hoc pairwise comparisons were performed with a Bonferroni-adjusted p-value threshold of < 0.016 to account for multiple comparisons. Categorical variables were compared using the chi-square test. The independent association between variables and pregnancy duration was assessed using multiple linear regression analysis. To assess multicollinearity among the independent variables, variance inflation factors (VIF) were calculated. VIF values below 5 were considered indicative of acceptable collinearity. Correlations were evaluated using Spearman’s rank-order correlation with a two-tailed test. For all other analyses, a p-value of < 0.05 was considered statistically significant.

## Results

A total of 346 pregnancies were included in the analysis, comprising 41 late preterm, 130 early term, and 175 full-term births. The mean maternal age, gestational age at sonographic assessment, duration of pregnancy, and birthweight for the entire cohort were 30.5 ± 4.87 years, 12.6 ± 0.6 weeks, 271 ± 10.5 days, and 3220 ± 447 g, respectively. The mean free β-hCG and PAPP-A levels were 38 ± 26.2 mIU/mL and 3.56 ± 2.63 mU/mL, respectively. The corresponding mean MoM values were 1.28 ± 0.84 for β-hCG and 1.08 ± 0.58 for PAPP-A, while the mean uterine artery Doppler pulsatility index (UtAD PI) was 1.54 ± 0.49.

Maternal characteristics and first-trimester screening parameters according to gestational age at birth are presented in Table 1. Maternal age was comparable among the groups (*p* = 0.138). However, nulliparity differed significantly, with a higher proportion observed in the full-term group compared to the late preterm group (65% vs. 41%, *p* = 0.007). Birthweight increased significantly with advancing gestational age (*p* = 0.002), with all pairwise comparisons remaining significant after Bonferroni correction (*p* < 0.001 for all).

**Table 1:** Maternal characteristics and first-trimester combined screening parameters according to gestational age at birth

	Late preterm	Early Term	Full Term	p	p1	p2	p3
N	41	130	175				
Maternal age	31.2 (26.5–35.2)	30.4 (27.8–33.6)	29.7 (26.7–33.1)	0.138	0.77	0.227	0.064
Nulliparity	17 (41)	77 (59)	115 (65)	0.025	0.074	0.007	0.229
Birthweight (gram)	2710 ± 558	3173 ± 425	3374 ± 322	0.002	< 0.001	< 0.001	< 0.001
Fetal sex				0.078	0.517	0.065	0.074
Male	17 (41)	61 (47)	101 (57)				
Female	24 (59)	69 (53)	74 (43)				
Free 𝛽-hCG (mIU/mL)	28.4 (18.4–57.5)	29.1 (19.5–42.3)	32.2 (21.8–50)	0.511	0.961	0.569	0.255
Free 𝛽-hCG MoM	1.07 (0.76–1.72)	0.98 (0.74–1.45)	1.04 (0.75–1.56)	0.607	0.445	0.807	0.383
PAPP-A (mU/mL)	2.44 (1.43–4.01)	2.75 (1.63–4.21)	3.22 (2.06–4.78)	0.030	0.51	0.036	0.032
PAPP-A MoM	0.83 (0.57–1.33)	0.92 (0.64–1.38)	1 (0.75–1.3)	0.192	0.587	0.097	0.200
Overall UtAD PI	1.59 ± 0.57	1.51 ± 0.5	1.55 ± 0.46	0.859	0.458	0.666	0.572
Sonographic GA (weeks)	12.6 (12.2–13.2)	12.5 (12.2–13.1)	12.6 (12.1–13.2)	0.668	0.642	0.896	0.377
Duration of pregnancy (days)	252 (248–256)	268 (264–270)	279 (275–283)	< 0.001	< 0.001	< 0.001	< 0.001

Data are presented as median (25th–75th percentile), mean ± SD, or N (%) according to the distribution of data

p: comparison across all groups; p1: late preterm vs. early term; p2: late preterm vs. full term; p3: early term vs. full term

Statistical significance was defined as p<0.05 for the global comparison (p) and p<0.016 for pairwise comparisons (p1,p2,p3) following Bonferroni correction

Abbreviations: *β-hCG* beta-human chorionic gonadotropin, *PAPP-A* pregnancy-associated plasma protein-A, *UtAD PI* uterine artery Doppler pulsatility index, *GA* gestational age, *MoM* multiples of the median, *SD* standard deviation

Fetal sex distribution did not differ significantly between groups (*p* = 0.078). Similarly, free β-hCG levels and MoM values were comparable (*p* = 0.511 and *p* = 0.607, respectively). Raw PAPP-A levels differed significantly across groups (*p* = 0.030), whereas PAPP-A MoM values were not (*p* = 0.192). No significant differences were observed in UtAD PI or gestational age at screening (*p* = 0.859 and *p* = 0.668, respectively). As expected, duration of pregnancy differed significantly across all groups (*p* < 0.001), with all pairwise comparisons remaining significant after correction.

Maternal characteristics, first-trimester β-hCG, uterine artery Doppler indices, birthweight, and duration of pregnancy stratified according to PAPP-A MoM levels (< 0.6 vs. ≥0.6) are presented in Table 2. Maternal age and fetal sex distribution were similar between groups (*p* = 0.207 and *p* = 0.193, respectively), whereas nulliparity was less frequent in the low PAPP-A group (47% vs. 63%, *p* = 0.025). Birthweight was significantly lower in pregnancies with PAPP-A MoM < 0.6 (3045 ± 535 g vs. 3259 ± 415 g, *p* < 0.001). Free β-hCG levels were also lower in this group (*p* = 0.013), although β-hCG MoM values were comparable (*p* = 0.069). UtAD PI was significantly higher in the low PAPP-A group (1.70 ± 0.46 vs. 1.50 ± 0.49, *p* = 0.023), while gestational age at screening did not differ (*p* = 0.111). Duration of pregnancy was significantly shorter in the low PAPP-A group (269 vs. 273 days, *p* = 0.001).

**Table 2:** Comparison of maternal characteristics, first trimester β-hCG and uterine artery Doppler indices, birthweight and duration of pregnancy according to PAPP-A MoM levels

	PAPP-A < 0.6 MoM	PAPP-A ≥0.6 MoM	*p*
*N*	63	283	
Maternal age (years)	30.6 (27.6–34.5)	32.5 (26.9–33.3)	0.207
Nulliparity	30 (47)	179 (63)	0.025
Birthweight (gram)	3045 ± 535	3259 ± 415	< 0.001
Fetal sex			0.193
Male	37 (59)	142 (50)	
Female	26 (41)	141 (50)	
Free 𝛽-hCG (mIU/mL)	28.2 (17–37.5)	32.2 (21–50.9)	0.013
Free 𝛽-hCG MoM	0.95 (0.69–1.35)	1.04 (0.75–1.62)	0.069
Overall UtAD PI	1.70 ± 0.46	1.50 ± 0.49	0.023
Sonographic GA (weeks)	12.5 (12.1–13.1)	12.5 (12.2–13.2)	0.111
Duration of pregnancy (days)	269 (264–274)	273 (267–280)	0.001

Data are presented as median (25th–75th percentile), mean ± SD, or N (%) according to the distribution of data

Abbreviations: *β-hCG* beta-human chorionic gonadotropin, *PAPP-A* pregnancy-associated plasma protein A, *UtAD PI* uterine artery Doppler pulsatility index, *GA* gestational age

To assess the robustness of the findings, sensitivity analyses were performed using PAPP-A MoM thresholds of 0.4 and 0.5. At the 0.4 MoM cut-off, median gestational duration was 265.5 days in the low PAPP-A group (*n* = 18) versus 273 days in the high PAPP-A group (*n* = 328); however, this difference did not reach statistical significance (*p* = 0.060), likely due to the limited sample size in the low PAPP-A group. At the 0.5 MoM cut-off, gestational duration was significantly shorter in the low PAPP-A group (268.5 days, *n* = 40) compared to the high PAPP-A group (273 days, *n* = 306; *p* = 0.004), consistent with the primary analysis. As the 0.6 MoM threshold yielded the highest statistical significance (*p* = 0.001) among the cut-offs evaluated, it was selected for the primary group comparison.

Comparisons of maternal characteristics, first-trimester biochemical markers, uterine artery Doppler indices, birthweight, and duration of pregnancy according to fetal sex and nulliparity are presented in Table 3.

**Table 3:** Comparison of first-trimester β-hCG, PAPP-A, uterine artery Doppler indices, birthweight, and duration of pregnancy stratified by fetal sex and nulliparity

	Fetal Sex		*p*	Nulliparity		*p*
	Female	Male		Nulliparous	Parous	
N	168	178		209	137	
Maternal age	30 (27.1–33.4)	30.1 (27.4–33.5)	0.918	28.6 (26.2–31.9)	32.3 (28.7–36.3)	< 0.001
Birthweight (gram)	3160 ± 473	3272 ± 416	0.021	3256 ± 414	3195 ± 483	0.233
Free 𝛽-hCG (mIU/mL)	33.5 (23.5–58.9)	28.5 (18.1–39.4)	< 0.001	31.7 (21.6–42.7)	31.1 (20–54.9)	0.931
Free 𝛽-hCG MoM	1.18 (0.81–1.92)	0.96 (0.67–1.37)	< 0.001	1.04 (0.75–1.46)	1.04 (0.75–1.66)	0.915
PAPP-A (mU/mL)	3.14 (1.82–4.65)	2.9 (1.85–4.34)	0.617	3.26 (2.08–4.77)	2.44 (1.53–3.93)	< 0.001
PAPP-A MoM	0.99 (0.7–1.36)	0.92 (0.65–1.31)	0.639	1 (0.73–1.44)	0.83 (0.61–1.15)	< 0.001
Overall UtAD PI	1.53 ± 0.48	1.56 ± 0.51	0.728	1.5 ± 0.44	1.62 ± 0.55	0.109
Sonographic GA (weeks)	12.5 (12.1–13.1)	12.5 (12.2–13.2)	0.656	12.5 (12.2–13.1)	12.5 (12.1–13.2)	0.302
Duration of pregnancy (days)	271 (263–278)	274 (268–280)	0.016	274 (268–280)	271 (262–278)	0.005

Data are presented as median (25th–75th percentile) and mean ± SD according to the distribution of data

Abbreviations: *β-hCG* beta-human chorionic gonadotropin, *PAPP-A* pregnancy-associated plasma protein A, *UtAD PI* uterine artery Doppler pulsatility index, *GA* gestational age

When stratified by fetal sex, maternal age was comparable between groups (*p* = 0.918). Male fetuses were associated with higher birthweight compared to females (3272 ± 416 g vs. 3160 ± 473 g, *p* = 0.021). In contrast, free β-hCG levels and MoM values were significantly higher in pregnancies with female fetuses (*p* < 0.001 for both). No significant differences were observed in PAPP-A levels, PAPP-A MoM, uterine artery Doppler indices, or gestational age at screening (*p* > 0.05 for all). Duration of pregnancy was slightly longer in male fetuses compared to females (274 vs. 271 days, *p* = 0.016).

When stratified by parity, maternal age was significantly higher in parous women compared to nulliparous women (*p* < 0.001). PAPP-A levels and PAPP-A MoM values were significantly higher in nulliparous women (*p* < 0.001 for both), whereas free β-hCG levels and MoM values were comparable between groups (*p* = 0.931 and *p* = 0.915, respectively). No significant differences were observed in uterine artery Doppler indices, birthweight, or gestational age at screening (*p* > 0.05 for all). Duration of pregnancy was significantly longer in nulliparous women compared to parous women (274 vs. 271 days, *p* = 0.005).

Spearman’s rank correlation analysis demonstrated a weak but statistically significant positive correlation between pregnancy duration and both raw PAPP-A levels (rs = 0.167, *p* = 0.002) and PAPP-A MoM (rs = 0.134, *p* = 0.013). In multiple linear regression analysis adjusting for nulliparity and fetal sex, PAPP-A MoM was found to be independently associated with pregnancy duration (B = 2.08, *p* = 0.034). Nulliparity (B = 2.96, *p* = 0.013) and fetal sex (B = 2.63, *p* = 0.022) also remained significant independent predictors. The overall model explained a modest proportion of the variance in gestational duration (R² = 0.056, Adjusted R² = 0.047). Multicollinearity diagnostics confirmed the independence of the predictors included in the regression model, with VIF values of 1.000 for fetal sex, 1.037 for nulliparity, and 1.037 for PAPP-A MoM, all well below the conventional threshold of 5. No significant correlations were observed between pregnancy duration and free β-hCG or β-hCG MoM (*p* = 0.077 and *p* = 0.298, respectively).

A weak but statistically significant positive correlation was also identified between PAPP-A MoM and birthweight (rs = 0.128, *p* = 0.018), whereas no significant correlations were observed for raw PAPP-A, free β-hCG, or β-hCG MoM (*p* = 0.107, *p* = 0.884, and *p* = 0.491, respectively).

**Fig. 2:**
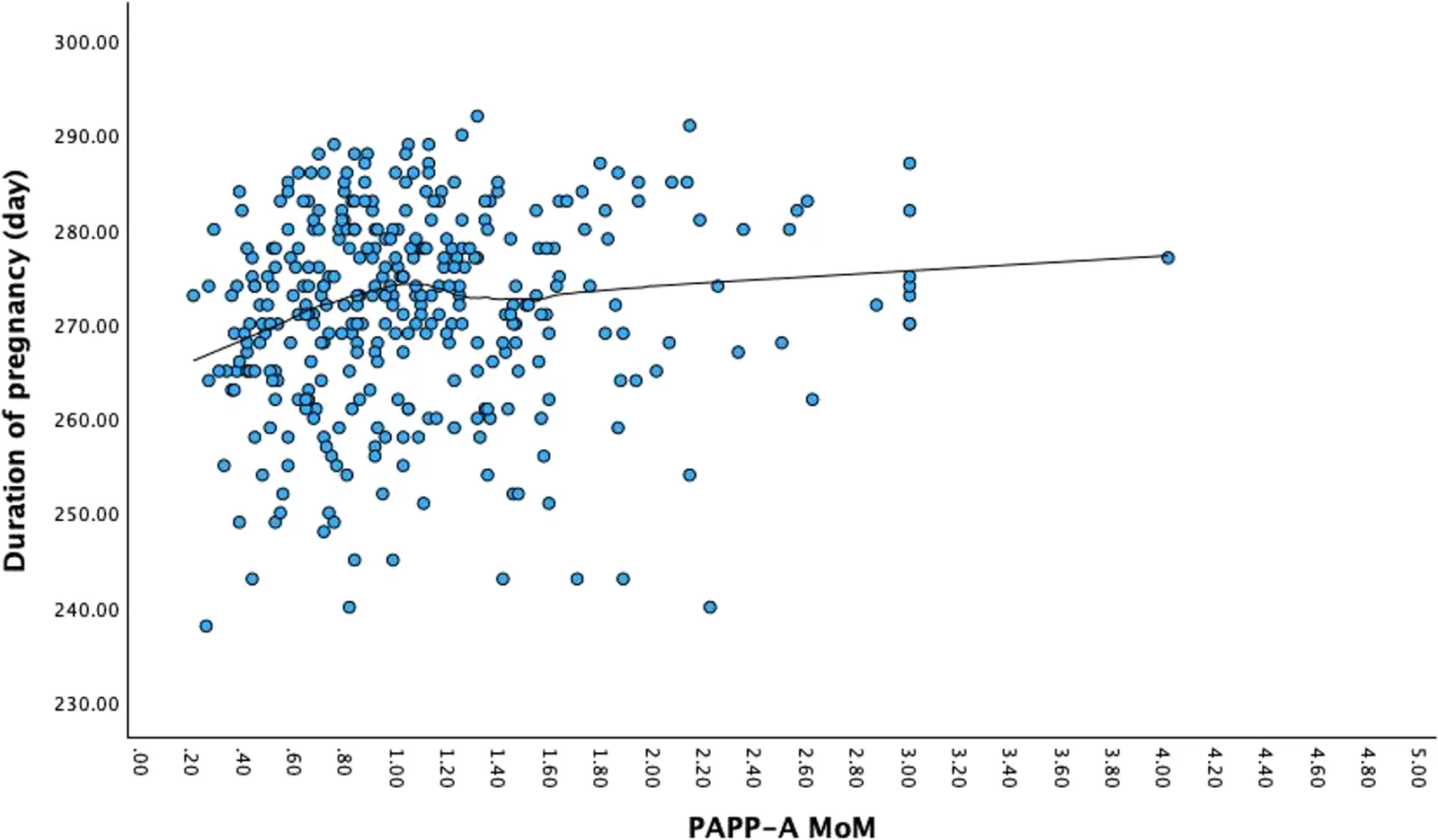
Scatter plot illustrating the relationship between PAPP-A MoM values and the mean duration of pregnancy (gestational age)

A scatter plot illustrating the relationship between PAPP-A MoM values and mean duration of pregnancy is presented in Fig. 2. Duration of pregnancy showed a gradual increase with rising PAPP-A MoM values, reaching a peak around 1.1 MoM, followed by a decline up to approximately 1.6 MoM. Beyond this point, a slight increase was observed; however, the number of pregnancies with very high MoM values was limited, precluding reliable interpretation of this pattern.

## Discussion

In the present study, the mean gestational length was 271 ± 10.5 days, which is approximately 9 days shorter than values reported in historical literature. An early study by Ahlfeld reported mean pregnancy durations of 284 days for first deliveries and 281 days for subsequent pregnancies, calculated from the last menstrual period (LMP) to delivery [11]. Subsequent studies have reported similar findings, with mean gestational durations ranging from 277 to 282 days based on LMP [12, 13]. In a large population-based study, Bergsø et al. analyzed 427,581 singleton births from national Swedish data and reported a mean gestational duration of 282 days for vaginal deliveries and 274 days for cesarean Sect [14].

In contrast, and consistent with our findings, a study from India reported a mean gestational duration of 272.1 ± 9 days among 1,094 women presenting in spontaneous labor [15]. Differences in study design and methodology likely account for these discrepancies. For instance, Bergsø et al. reported a postterm pregnancy rate of approximately 10%, defining postterm pregnancy as a duration beyond 294 days, and included all singleton births, thereby reflecting overall population characteristics [14]. In contrast, our study applied strict inclusion criteria to better isolate the association between PAPP-A levels and gestational duration, excluding medically indicated inductions, including those performed for postterm pregnancies at or beyond 41 weeks of gestation. This selection approach may partly explain the shorter mean gestational duration observed in our cohort. Similarly, the inclusion of only women presenting in spontaneous labor may account for the concordance between our findings and those reported by Bhat et al. [15].

In the present study, nulliparity was observed significantly more frequently in the full-term group than in the late preterm group. This finding contrasts with results from a nationwide study by Koullali et al., which identified nulliparity as an independent risk factor for spontaneous preterm birth, associated with nearly a twofold increased risk (OR 1.95, 95% CI 1.89–2.00) [16]. However, this discrepancy is likely attributable to the selection criteria applied in our cohort. Specifically, the exclusion of medically indicated deliveries and elective cesarean sections—implemented to focus on the timing of spontaneous labor—may have influenced the distribution of nulliparous women across gestational age groups. Therefore, our findings should be interpreted in the context of the study design rather than as contradictory to established epidemiological evidence.

The median raw PAPP-A levels increased progressively from the late preterm to the full-term group, and the differences between groups were statistically significant. Similarly, median PAPP-A MoM values showed a gradual increase from 0.83 in the late preterm group to 1.0 in the full-term group; however, this trend did not reach statistical significance, possibly due to the relatively small sample size in the subgroups.

In contrast to PAPP-A, raw free β-hCG levels and MoM values did not differ significantly across gestational age groups and showed no significant correlation with gestational duration. This finding is consistent with the distinct biological roles of these two markers. While PAPP-A modulates IGF bioavailability and directly influences trophoblastic invasion and placental development, free β-hCG primarily reflects trophoblast activity and corpus luteum support in early pregnancy. Its levels peak around 10–12 weeks of gestation and decline thereafter, rendering it a less informative marker of ongoing placental functional capacity later in pregnancy [17]. The absence of a significant association between β-hCG MoM and gestational duration in the present study is therefore biologically plausible and consistent with prior literature suggesting that β-hCG is a weaker predictor of placentation-related adverse outcomes compared to PAPP-A.

To further evaluate the association, patients were stratified into two groups according to PAPP-A MoM levels (< 0.6 and ≥ 0.6). Nulliparity was significantly more frequent in the group with PAPP-A MoM ≥ 0.6. Consistent with this finding, Illescas et al. reported that both mean and median PAPP-A MoM values were significantly higher in first pregnancies compared to subsequent pregnancies [18]. As expected, mean UtAD PI values were significantly lower in the PAPP-A MoM ≥ 0.6 group. Herraiz et al. previously demonstrated a significant inverse relationship between mean UtAD PI and PAPP-A levels in pregnancies at high risk for preeclampsia [19]. Both parameters are considered indirect markers of placental function; adequate PAPP-A levels are associated with enhanced trophoblastic invasion of the spiral arteries, resulting in reduced uterine vascular resistance and, consequently, lower UtAD PI values.

In our cohort, birthweight was significantly higher and pregnancy duration was significantly longer in the PAPP-A MoM ≥ 0.6 group. Consistent with these findings, previous studies have shown that lower PAPP-A MoM values are associated with an increased risk of small-for-gestational-age (SGA) fetuses; however, the predictive value of first-trimester PAPP-A for identifying SGA at term remains limited [9, 10, 20, 21]. Accordingly, in the present study, pregnancy duration was significantly longer in the PAPP-A MoM ≥ 0.6 group compared to the < 0.6 group (273 vs. 269 days).

Fetal sex and nulliparity were identified as factors influencing pregnancy duration. Male fetuses were associated with higher birthweight and longer gestational duration, while nulliparous women had longer pregnancies and higher PAPP-A MoM values. In multivariable regression analysis, PAPP-A MoM, nulliparity, and fetal sex emerged as independent predictors of pregnancy duration. The literature regarding the effect of parity on gestational duration remains inconsistent. A large population-based cohort study reported a small but statistically significant difference, with nulliparous pregnancies being approximately 0.3 days longer [14], whereas another study did not demonstrate a significant effect of parity or fetal sex on gestational duration [22].

When interpreted within the framework of the EGG hypothesis, these findings suggest that improved early placental function—reflected by higher PAPP-A levels—may contribute to prolonged gestation. More efficient placental development may enhance maternal–fetal energy transfer, thereby delaying the point at which fetal metabolic demands exceed maternal supply. This, in turn, may postpone the activation of metabolic signals involved in the initiation of parturition.

The present study has several strengths. First, the study population was carefully selected using strict inclusion and exclusion criteria, allowing for the evaluation of gestational duration in a relatively homogeneous cohort of women presenting in spontaneous labor. By excluding medically indicated deliveries, elective cesarean sections, and pregnancies complicated by major maternal or fetal conditions, we were able to minimize confounding factors that could artificially influence the timing of birth. Second, gestational age was determined using first-trimester crown–rump length measurements, which improves the accuracy of pregnancy dating compared to last menstrual period-based estimates. Third, the combined assessment of biochemical markers and uterine artery Doppler indices provides a comprehensive evaluation of early placental function.

However, several limitations should be acknowledged. The retrospective and single-center design may limit the generalizability of the findings and introduces the potential for selection bias. In particular, the exclusion of postterm inductions may have contributed to the shorter mean gestational duration observed in our cohort. Additionally, although key confounders such as nulliparity and fetal sex were accounted for in the regression analysis, residual confounding cannot be excluded. Maternal BMI, smoking status, and socioeconomic status are recognized determinants of gestational duration that were not separately evaluated. It should be noted that MoM values for PAPP-A and free β-hCG were adjusted for maternal BMI, smoking status, maternal age, and sonographic gestational age using validated prenatal screening software. While this adjustment partially accounts for these variables, it may also obscure direct associations between these characteristics and gestational duration that operate through pathways independent of placental biomarker levels. Gestational duration is a complex, multifactorial process influenced by a wide range of determinants, including genetic predisposition, fetal characteristics, placental development, maternal physiology, and even paternal factors. These variables were not comprehensively captured in the present study. Finally, the effect size of PAPP-A MoM on gestational duration was relatively modest, and the overall model explained only 4.7% of the variance in gestational duration, underscoring that first-trimester PAPP-A MoM alone has limited clinical utility for predicting the timing of delivery.

In conclusion, the findings of this study suggest that higher first-trimester PAPP-A MoM levels are independently associated with longer gestational duration, even after adjustment for fetal sex and parity. This association may reflect the role of early placental function in regulating the timing of parturition. Nevertheless, gestational duration remains a complex biological trait. While PAPP-A may serve as an early indicator of placental function, its role in predicting the timing of delivery appears limited when considered in isolation. Future prospective studies integrating biochemical, biophysical, and genomic data in larger and more diverse cohorts are needed to better elucidate the mechanisms underlying the timing of birth and to improve the prediction of gestational duration.

## Data Availability

The data used to support the findings of this study are available from the corresponding author upon reasonable request. We did not use artificial intelligence (AI) or AI-assisted technologies at any stage of our article.
